# Rational Design of Thermosensitive Hydrogel to Deliver Nanocrystals with Intranasal Administration for Brain Targeting in Parkinson's Disease

**DOI:** 10.34133/2021/9812523

**Published:** 2021-11-19

**Authors:** Yun Tan, Yao Liu, Yujing Liu, Rui Ma, Jingshan Luo, Huijie Hong, Xiaojia Chen, Shengpeng Wang, Chuntai Liu, Yi Zhang, Tongkai Chen

**Affiliations:** ^1^Hunan Provincial Key Laboratory of Micro & Nano Materials Interface Science, College of Chemistry and Chemical Engineering, Central South University, Changsha 410083, China; ^2^Science and Technology Innovation Center, Guangzhou University of Chinese Medicine, Guangzhou 510405, China; ^3^State Key Laboratory of Quality Research in Chinese Medicine, Institute of Chinese Medical Sciences, University of Macau, Macau 999078, China; ^4^Key Laboratory of Materials Processing and Mold, Ministry of Education, Zhengzhou University, Zhengzhou 450002, China

## Abstract

Mitochondrial dysfunction is commonly detected in individuals suffering from Parkinson's disease (PD), presenting within the form of excessive reactive oxygen species (ROS) generation as well as energy metabolism. Overcoming this dysfunction within brain tissues is an effective approach to treat PD, while unluckily, the blood-brain barrier (BBB) substantially impedes intracerebral drug delivery. In an effort to improve the delivery of efficacious therapeutic drugs to the brain, a drug delivery platform hydrogel (MAG-NCs@Gel) was designed by complexing magnolol (MAG)-nanocrystals (MAG-NCs) into the noninvasive thermosensitive poly(*N*-isopropylacrylamide) (PNIPAM) with self-gelation. The as-prepared MAG-NCs@Gel exhibited obvious improvements in drug solubility, the duration of residence with the nasal cavity, and the efficiency of brain targeting, respectively. Above all, continuous intranasal MAG-NCs@Gel delivery enabled MAG to cross the BBB and enter dopaminergic neurons, thereby effectively alleviating the symptoms of MPTP-induced PD. Taking advantage of the lower critical solution temperature (LCST) behavior of this delivery platform increases its viscoelasticity in nasal cavity, thus improving the efficiency of MAG-NCs transit across the BBB. As such, MAG-NCs@Gel represented an effective delivery platform capable of normalizing ROS and adenosine triphosphate (ATP) in the mitochondria of dopaminergic neurons, consequently reversing the mitochondrial dysfunction and enhancing the behavioral skills of PD mice without adversely affecting normal tissues.

## 1. Introduction

Parkinson's disease (PD) is one of the most severe neurodegenerative diseases which affects approximately 2% of individuals over the age of 65, resulting in motor and nonmotor dysfunction because of the degenerating the dopaminergic neurons in the substantia nigra [1–3]. PD incidence among younger individuals has been rising in recent years and therefore represents a major threat to global public health [4]. At present, drug-based therapeutics are the primary tools used to treat PD, with dopamine receptor agonists, monoamine oxidase B inhibitors, and the dopamine precursor levodopa being the main treatments [5, 6]. These pharmacological agents can slacken disease progression, alleviate symptoms, and thereby improve patient quality of life. However, most of known drugs are incapable of efficiently penetrating the blood-brain barrier (BBB) and cause substantial systemic adverse effects, limiting their clinical utility [7, 8].

The intranasal delivery of PD drugs is often considered to be preferable owing to its ability to decrease systemic drug delivery while by passing the selectivity of the BBB to directly transport pharmacological agents into the brain via the trigeminal and olfactory systems that link the external environment directly to the central nervous system (CNS) [9–11]. In recent years, natural products, as promising resources with sustainable and low toxicity, have attracted considerable attentions in the treatment of PD by nasal administration [12–14]. In addition, by evading first-pass hepatic metabolism, this approach also reduces the rates of drug degradation. Several nanoparticle-based strategies, such as solid lipid nanoparticles [15, 16], nanoemulsions [17], liposomes [18], polymeric micelles [19, 20], and cyclodextrin derived [21] have been used to achieve efficient intranasal drug delivery to date. Nanocrystal- (NC-) based delivery platforms are particularly promising in this therapeutic context, as they consist of small crystals (<500 nm) stabilized with surfactants or polymeric steric stabilizers [22, 23]. The NC delivery platforms enable researchers to achieve targeted therapy and efficient treatment and contribute to transform the balance from side effect therapy to a beneficial one [24, 25]. After production, these NCs can be loaded with drugs of interest at high levels without organic solvents and remain highly stable and less toxic than other nanoscale drug delivery platforms. Despite those innovative advances in fabrication, structural characterization, and formation mechanism [26, 27], as well as the tests of in vitro therapeutic efficacy [28], the clinical application of most NCs has been hindered by their poor solubility and strong self-aggregation tendency [29]. To establish a rational NC-based drug carrier system within the nasal cavity still remains a great challenge.

Hydrogel function is treated as ideal drug carriers owing to their ability to overcome a range of limitations [30–33]. In particular, thermosensitive hydrogels can be prepared and loaded with drugs under relatively mild conditions, exhibiting thermally controlled drug loading/unloading behavior when applied to the nasal mucosa in the form of an injectable preparation owing to their shear thinning properties [34–36]. Poly(N-isopropylacrylamide) (PNIPAM) hydrogel is a typical thermosensitive hydrogel with reversible lower critical solution temperature (LCST) behavior owing to its temperature-dependent hydrophilicity and hydrophobicity [37–39]. The LCST of PNIPAM hydrogels is typically approximately 32°C [40]. The exposure of these hydrogels to warmer temperatures within the nasal cavity can result in increased hydrogel viscoelasticity, thereby reducing the rate of mucociliary clearance and ensuring gradual sustained drug release. Such a thermosensitive hydrogel delivery system has offered great potential to improve drug potency and to absorb significant amounts of water or biological fluids [41, 42]. Meanwhile, these hydrogels are also both biocompatible and biodegradable, making them ideal tools for clinical application.

**Figure 1:**
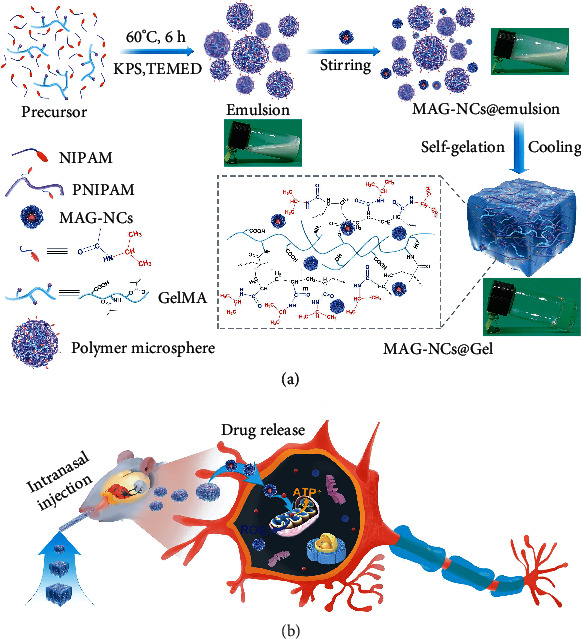
Synthesis and application of MAG-NCs@Gel. (a) Schematic diagram of MAG-NCs@Gel preparation and (b) the targeting treatment of PD via the intranasal injection of the hydrogel with release NCs.

Inspired by abovementioned pioneering works and integrating our previous experience [8, 43, 44], we attempted to employ the hydroxylated biphenol derived from the ‘Houpo' herb (Magnolia officinalis) known as magnolol (MAG) as a model drug, as it is commonly studied to treat PD [45, 46]. To improve the solubility, stability, and bioavailability characteristics of MAG, PVP-K30 was used to stabilize MAG-containing NCs (MAG-NCs). Further, in order to construct the MAG-NCs@emulsion, the as-developed MAG-NCs were then uniformly dispersed into an emulsion of thermosensitive PNIPAM, which was obtained by in situ free radical polymerization of precursor (Figure 1(a)). Following, as a self-gelation process, it is wonderful that the MAG-NCs@emulsion polymer changed from a fluid to a solid-like state during natural cooling, yielding the transparent polymer network MAG-NCs@Gel (Movie S1). This MAG-NCs@Gel preparation was then intranasally injected into experimental model mice, whereupon it was able to penetrate the BBB to achieve effective drug release, thereby targeting mitochondria within neurons to reduce ROS generation and increase ATP levels as a treatment for PD (Figure 1(b)). The thermosensitive hydrogel will happen in phase transition and exhibit the characteristics of opacity and volume shrinkage, when the temperature is higher than the LCST of the hydrogel [47, 48]. According to the relationship between the transparency of the hydrogels and the temperature, the LCST of MAG-NCs@Gel and blank gel have been determined at about ~32.2°C and 32.4°C, respectively (Figure S1). Since the body temperature (of murine and rats) is higher than the LCST of MAG-NCs@Gel, the volume of MAG-NCs@Gel will shrink to some extent in these animals. In addition, storage modulus (*G*′) and loss modulus (*G*^″^) of MAG-NCs@Gel increased with rising temperature (Figure S2), indicating that MAG-NCs@Gel with slightly contracted volume exhibited better viscoelasticity in the nasal cavity than the external body. Therefore, these rationally designed MAG-NCs@Gel preparations were able to overcome limited MAG solubility within aqueous environments, while simultaneously achieving prolonged MAG residence in the nasal cavity. Importantly, encapsulated MAG-NCs were able to efficiently transit across the BBB, thus improving their cerebral bioavailability and enabling the targeted treatment of PD.

## 2. Results

### 2.1. Characterization of MAG-NCs and MAG-NCs@Gel

**Figure 2:**
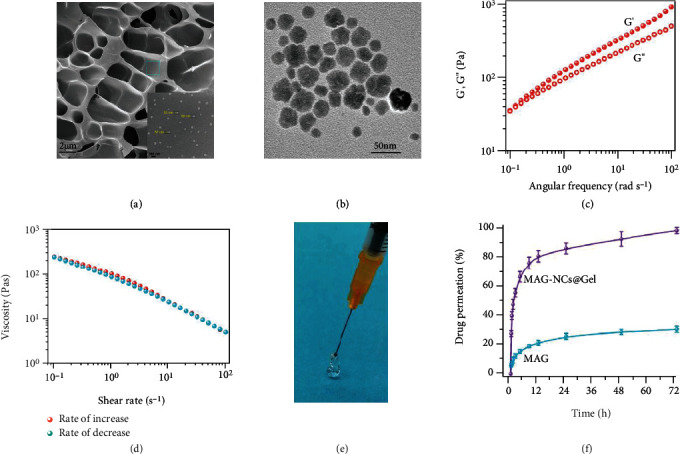
Structure characterization and performance measurement of MAG-NCs@Gel. SEM (a) and TEM (b) images of MAG-NCs@Gel preparations. (c) The dependence of *G*′ and *G*^″^ of MAG-NCs@Gel on the angular frequency. (d) The association between MAG-NCs@Gel viscosity and shear rate. (e) Optical image of MAG-NCs@Gel injection. (f) Drug ex vivo nasal mucosa permeation curves as a function of time.

The dynamic light scattering (DLS) was initially used to assess the hydrated particle size of MAG-NCs, which exhibited a mean diameter of 81.57 ± 1.48 nm and the mean particle distribution index (PDI) of 0.11 ± 0.02 (Figure S3a), and the mean hydrated MAG-NC particle size for storage one month was still 81-84 nm with PDI of 0.1-0.2 (Figure S3b), these indicating that MAG-NC remained stable for a month or more. Relative to the sizes of hydrated particles detected via DLS, TEM imaging suggested MAG-NCs to be somewhat smaller about 30 nm (Figure S3c), because TEM images revealed the true size of the drug NCs without accounting for the PVP-K30 coating or the electric double layer. To further confirm MAG-NC stability, TEM images of MAG-NCs were captured following storage for two weeks, revealing that particles were still well dispersed, neither agglomeration nor dissociation (Figure S3d). Meanwhile, SEM images of freeze-dried MAG-NCs@Gel indicated the uniform distribution of MAG-NCs within honeycomb polymer network, and the size of MAG-NCs was about 50-70 nm (Figure 1(a)). The SEM of the blank hydrogel shows the homogeneous polymer network, except without MAG-NCs (Figure S4). In addition, those MAG-NCs were also clearly observed in SEM images of MAG-NCs@Gel following a two-week storage at room temperature, suggesting that MAG-NCs@Gel preparations are highly stable (Figure S5). Besides, TEM images of MAG-NCs@Gel preparations show the size of MAG-NCs to range from 20 to 30 nm, which was closed to that of the MAG-NC dispersion (Figure 1(b)). FT-IR spectra of MAG-NCs@Gel displayed the presence of absorption peaks at 1635 cm^−1^ (C=O), 1530 cm^−1^ (N-H of amide), 1455 cm^−1^ (C-H), and 3291 cm^−1^ (N-H stretching vibration peak of GelMA) (Figure S6) [49]. Owing to the very low MAG-NC content in these samples (<0.2 wt%), no characteristic peak of MAG-NCs was detectable. Rheological studies were then performed exploring the viscoelasticity and injectability of MAG-NCs@Gel [50, 51]. Based upon the dependence of the *G*′ and *G*^″^ of MAG-NCs@Gel on the oscillation strain (Figure S7), the reversible gel state to flow state transition was determined to occur at ~80% strain (gel state: *G*′ > *G*^″^ and flow state: *G*′ < *G*^″^) [52]. Similarly, the gel-flow state transition of the blank hydrogel also occurred at 80% strain. Step-strain measurements with 5 times cycle revealed that *G*′ was higher than *G*^″^ at low strain (1%), whereas at higher strain levels (200%), *G*′ was lower than *G*^″^ (Figure S8). Those results suggested that the MAG-NCs@Gel exhibited self-healing properties [53]. In frequency sweep analyses, the *G*′ and *G*^″^ of MAG-NCs increased with increasing angular frequency, and *G*′ was always greater than *G*^″^, so that gel characteristics were observed across the entire frequency range (Figure 2(c)). Relative to MAG-NCs@Gel, the *G*′ and *G*^″^ of the blank hydrogel were smaller, although they were similarly dependent upon angular frequency (Figure S9). This suggests that interactions between MAG-NCs and polymer networks can enhance MAG-NCs@Gel viscoelasticity to some degree. MAG-NCs@Gel shear thinning was assessed based upon the association between the viscosity and shear rate (Figure 2(d)). The viscosity declines as the shear rate increases, suggesting that this hydrogel exhibits promising injectable properties (Figure 2(e), Movie S2) [54, 55]. In order to obtain the release performance of MAG-NCs@Gel polymer network for MAG-NC_S_, *in vitro* nasal mucosa permeation assays were carried out, the results indicated that ~100% of MAG-NCs were released from MAG-NCs@Gel within 72 h (Figure 2(f)), in which the permeation efficiency was higher than the MAG group (~30.7%) and more durable release time as compared to the MAG-NCs group (Figure S10). These results suggested that MAG-NCs@Gel hydrogel loading significantly improved in vitro release and prolonged the overall drug release duration through the interaction between polymer and MAG-NCs to construct the suitable network structure.

### 2.2. In Vitro Neuroprotective Efficacy of MAG-NCs

**Figure 3:**
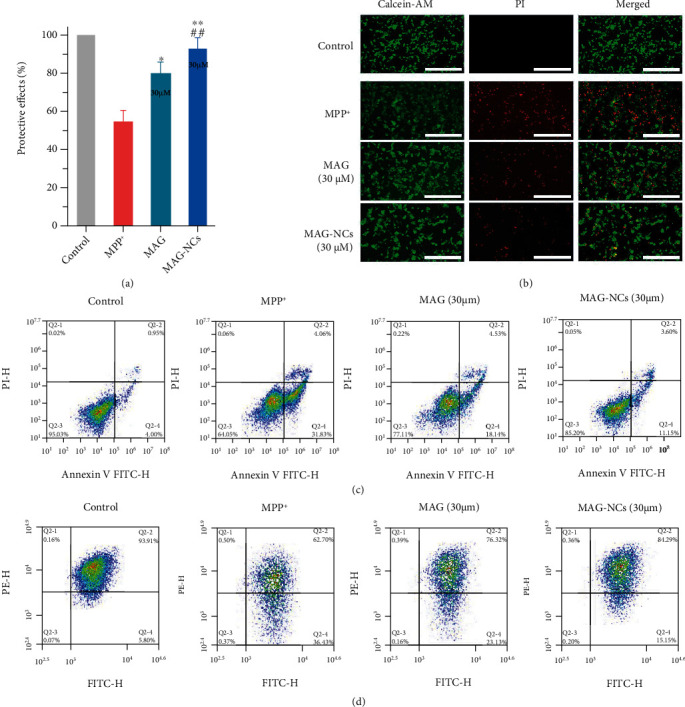
Protective effects of MAG-NCs. (a) Protective effects of MAG (30 *μ*M) and MAG-NCs (30 *μ*M) against MPP^+^-induced cell death and cytotoxicity. ^∗^*p* < 0.05 and ^∗∗^*p* < 0.01 vs. the MPP^+^ group. ^#^*p* < 0.05 and ^##^*p* < 0.01 vs. the MAG group at the same concentration. (b) Confocal laser scanning microscopy (CLSM) images of SH-SY5Y cells in the indicated treatment groups, with live and apoptotic cells being stained green (Calcein-AM) and red (propidiumiodide (PI)), respectively. Flow cytometry (c) and mitochondrial membrane potential (d) analysis of Annexin V FITC-H/PI-stained SH-SY5Y cells within the demonstrated treatment groups. Scale bars: 100 *μ*m.

To assess the neuroprotective properties of MAG and MAG-NC preparations within the context of MPP^+^-induced cytotoxicity, SH-SY5Y cell survival following treatment with these preparations was assessed via MTT assay [56]. At concentrations below 160 *μ*M, no significant cytotoxicity was observed for MAG or MAG-NCs (Figure S11). Meanwhile, neuroprotection was evaluated by treating SH-SY5Y cells with MAG or MAG-NCs (30 *μ*M). Cells treated with MPP^+^ (2 mM) exhibited a survival rate of just 54.1% compared to the control group (100% cell survival rate), whereas cells treated with MAG and MAG-NCs at the 30 *μ*M concentration exhibited respective viability values of 79.1% and 92.8% (Figure 3(a)). Even treatment with lower MAG and MAG-NC concentrations (7.5 *μ*M or 15 *μ*M) was associated with improved cell survival relative to the MPP^+^ group, although these effects were not as robust as those following treatment at the 30 *μ*M dose (Figure S12). Notably, the neuroprotective efficacy of MAG-NC preparations was superior to that of equivalent MAG concentrations. Consistently, CLSM images of SH-SY5Y cells stained implementing AM-PI (green-red) were prepared, with red and green fluorescence, respectively, corresponding to apoptotic and viable cells (Figure 3(b)). While control samples exhibited no evidence of cellular apoptosis, significant neurotoxic cell death was evident in the MPP^+^ model group, whereas both the 30 *μ*M MAG-NCs and 30 *μ*M MAG groups exhibited significant increases in the proportion of live cells, with this effect being more pronounced in the MAG-NCs group as compared to the MAG group. Flow cytometry analyses were further performed to quantify the neuroprotective effects of these preparations (Figure 3(c)), revealing respective frequencies of initial and tardy apoptotic cells in the MAG and MAG-NCs groups of 22.6% and 14.8%, with these rates being higher than those in the control group (~5%), but much lower than those in the MPP^+^ group (~36.9%). Mitochondrial membrane potential measurements also yielded similar results (Figure 3(d)). Mitochondria are the main place for ATP production and an important organelle to promote cell energy conversion. The decrease of mitochondrial membrane potential is a landmark event in the early stage of apoptosis. The percentage of apoptotic cells in the control, MPP^+^, MAG, and MAG-NCs groups was 5.86%, 36.43%, 23.1%, and 15.1%, respectively, confirming that MAG-NCs were associated with superior antiapoptotic efficacy.

### 2.3. Plasma and Brain Pharmacokinetics

MAG pharmacokinetic studies were performed in rats at a 1.0 mg/kg dose. In plasma pharmacokinetic analyses (Figure S13a), intranasally administered MAG-NCs@Gel was associated with sustained MAG detection in the plasma over 12 h postadministration, whereas plasma MAG levels in rats intravenously injected with MAG were only detectable in the first 3 hours (Table S1), and MAG-NC treatment prolonged the MAG circulation to the 6 hours. The MAG-NCs@Gel group exhibited a MAG *T*_max_ of 3.50 ± 0.55 h and *C*_max_ of 25.13 ± 3.87 ng/mL, whereas for MAG-NCs these values were 0.083 h and 128.87 ± 18.06 ng/mL, respectively (Table S2), indicating that intranasal MAG-NCs@Gel administration is associated with sustained and controlled drug release. Furthermore, the *T*_1/2_ of the MAG-NCs@Gel group (3.05 ± 0.41 h) was over 2-fold higher than that in both the MAG and MAG-NCs groups, consistent with the prolongation of plasma release time for MAG-NCs loaded into PNIAM hydrogel as compared to free MAG-NCs. Consistently, 10.14-fold and 3.34-fold increase in the MRT_0−*t*_ value for MAG were detected in rats in the MAG-NCs@Gel group (5.68 ± 0.47 h) relative to the MAG_*i.v*._ group (0.56 ± 0.15 h) and MAG-NCs group (1.70 ± 0.22 h), respectively. Furthermore, the AUC_0−*t*_ in MAG-NCs@Gel-treated animals was 302.07 ± 29.86 ng h mL^−1^, which was 7.47 times, 5.92 times, and 3.38 times higher than that observed for rats in the MAG*_i.v._* group (40.43 ± 4.17 ng h mL^−1^), MAG*_i.n._* (50.97 ± 6.09 ng h mL^−1^), and MAG-NCs group (89.27 ± 10.23 ng h mL^−1^). These results thus indicated that MAG-NCs improved the bioavailability of MAG, and MAG-NCs loaded with a thermosensitive hydrogel may be associated with better gradual MAG release, protecting against rapid loss of drug and facilitating effective drug release.

Direct delivery of medications to the brain is one of the most promising approaches to effectively treating PD. Therefore, drug pharmacokinetics within the brain is a key consideration when evaluating potential PD therapeutics. The association between MAG concentrations and time in the brain was thus assessed following its intranasal or intravenous administration (Figure S13b), and related pharmacokinetic parameters were calculated. The *T*_max_ value in animals in the MAG-NCs@Gel group (4.67 ± 1.03 h) was 9.34 times and 7.4 times that of animals in the MAG_*i.v*._ group (0.5 ± 0 h) and MAG-NCs group (0.63 ± 0.31 h), respectively. The *C*_max_ for the MAG-NCs@Gel group was also markedly increased relative to the MAG_*i.v*._, MAG*_i.n._*, and MAG-NCs groups (131.75 ± 10.21 ng/g vs. 22.85 ± 3.94, 33.01 ± 4.03, and 34.76 ± 3.19 ng/g). The *T*_1/2_ (11.51 ± 1.42 h) and *T*_max_ of the MAG-NCs@Gel group were also longer in the brain relative to the plasma, suggesting that the majority of administered MAG was effectively absorbed within the brain. The AUC_0−*t*_ in the MAG-NCs@Gel group was 2555.69 ± 336.17 ng h g-1, with this value being 27.4-fold higher than that in the MAG-NCs group (93.24 ± 10.96 ng h g^−1^). In summary, these results suggested that continuous drug delivery and effective BBB penetration were achieved in the MAG-NCs@Gel group by utilizing controlled-release MAG-NCs encapsulated within a PNIPAM hydrogel. These data indicated that MAG-NCs@Gel with DTE% and DTP% values of 809.98% and 87.65% were able to efficiently deliver MAG to the brain.

### 2.4. Treatment of MPTP-Induced Behavioral Deficits

**Figure 4:**
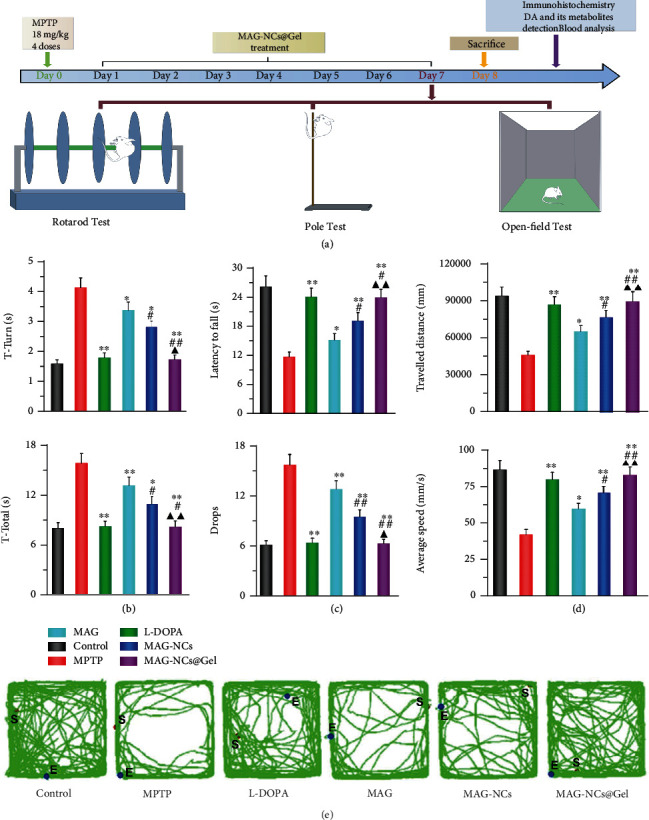
Behavioral analysis. (a) Schematic overview of the comprehensive experimental procedure for the MAG-NCs@Gel treatment of MPTP-induced PD. Murine behavioral analyses were conducted: (b) the rotarod test: *T*-turn (A) and *T*-total (B), (c) the pole test: latency to fall (A) and drops (B), and (d) the open field test: average travel distance (A) and speed (B). (e) Representative activity paths for mice in the indicated groups as measured via digital tracking (red: starting position; blue: ending position). ^∗^*p* < 0.05 and ^∗∗^*p* < 0.01 vs. the MPTP group. ^#^*P* < 0.05 and ^##^*P* < 0.01 vs. the MAG group. ^▲^*p* < 0.05 and ^▲▲^*p* < 0.01 vs. the MAG-NCs group.

To confirm the satisfactory therapeutic efficacy of MAG-NCs@Gel as a treatment for the behavioral deficits of MPTP-induced mice, a series of comprehensive experiments was performed (Figure 4(a)). Initially, MPTP was administered for mice (18 mg kg^−1^), resulting in significant motor behavioral deficits [57]. The effects of intranasal MAG-NCs@Gel administration on these deficits were then assessed, with mice being examined using the rotarod, pole, and open field tests seven days later. On day eight of this study, mice were then euthanized, and immunohistochemical staining for DA and its metabolites, blood analyses, and tests of hepatic and renal functionality were conducted. MPTP induces motor symptoms including impaired coordination and balance, bradykinesia, and diminished spontaneous motor performance. In rotarod tests, mice treated with MAG or L-DOPA exhibited significant improvements relative to MPTP-treated model mice (Figure 4(b)), with the values of time to reach the bottom (*T*-total) and time to turn (*T*-turn) (8.35 ± 0.71 s and 1.73 ± 0.15 s) in the MAG-NCs@Gel group being lower than those in the MAG group (3.37 ± 0.27 s and 13.31 ± 1.01 s) and the MAG-NCs group (2.81 ± 0.20 s and 11.08 ± 0.94 s), consistent with the optimal therapeutic efficacy of MAG-NCs@Gel preparations. Relative to other MAG treatments, the MAG-NCs@Gel group exhibited similar improvements in the pole test (Figure 4(c)), wherein MAG-NCs@Gel-treated mice exhibited prolonged fall latency and a reduced number of drops. There was also a significant increase in average travel distance and speed in the MAG-NCs@Gel group relative to other experimental groups in the open field test (Figures 4(d) and 4(e)). In summary, these behavioral tests indicated that MAG-NCS@Gel treatment was associated with excellent therapeutic efficacy as a treatment for MPTP-induced motor dysfunction, which was comparable to that detected in mice treated with L-DOPA.

### 2.5. The Prevention of TH^+^ Neuron Loss and Reversal of Mitochondrial Dysfunction

**Figure 5:**
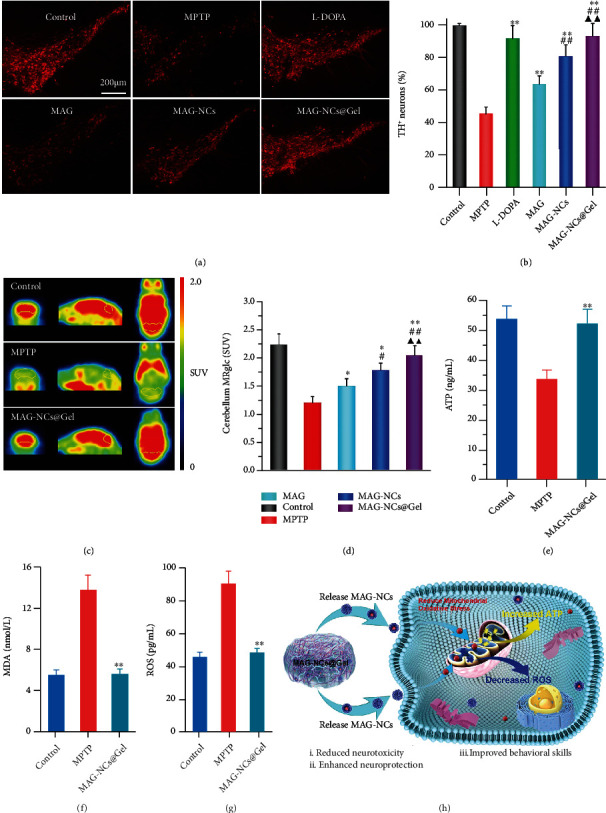
Targeted treatment of PD with MAG-NCs@Gel. (a) Images of TH^+^ immunofluorescent-stained sections. (b) Assessment of TH^+^ neurons in the substantia nigra. (c) ^18^F-FDG PET images of brain anatomy in the coronal, sagittal, and transverse directions. (d) ^18^F-FDG uptake values in the left striatum for the indicated groups. Mitochondrial functional parameters detecting in the midbrain, including (e) ATP levels, (f) MDA levels, and (g) ROS levels. (h) A schematic overview of the mechanistic treatment of mitochondrial dysfunction using the MAG-NCs@Gel controlled release platform. ^∗^*p* < 0.05 and ^∗∗^*p* < 0.01 vs. the MPTP group. ^#^*p* < 0.05 and ^##^*p* < 0.01 vs. the MAG group. ^▲^*p* < 0.05 and ^▲▲^*p* < 0.01 vs. the MAG-NCs group.

MPTP-induced neurotoxicity results in the loss or death of TH^+^ neurons in the midbrain substantia nigra together with reductions in levels of homovanillic acid (HVA) and striatal DA, 3,4-dihydroxyphenylacetic acid (DOPAC). Fluorescent images of TH^+^ neurons clearly emphasized the protective influences of MAG-NCs@Gel treatment against MPTP-induced neurotoxicity, with these results being better than those in the MAG or MAG-NCs groups (Figure 5(a)). Notably, the protective efficacy in the MAG-NCs@Gel group and the L-DOPA group were similar, with respective protection levels that were 92.94 ± 7.66% and 91.59 ± 7.84% of those in the control group, respectively (Figure 4(b)). In addition, MPTP treatment rapidly reduced striatal DA, DOPAC, and HVA levels, whereas all experimental treatments significantly increased these levels. The protective effect on neurons of MAG-NCs had been confirmed in the in vitro experiment. Importantly, these improvements were most robust in the MAG-NCs@Gel group relative to other MAG treatment groups (Figure S14), indicating that this drug release platform was able to effectively protect against MPTP-induced TH^+^ neuron death and loss while improving the DA and DA metabolites levels within the striatum.

One of the most valuable noninvasive imaging tools for monitoring changes in the cerebral blood flow in vivo is PET, and it is thus widely used to diagnose PD and other neurodegenerative diseases, including those where in neuronal dysfunction precedes structural change. In such analyses, ^18^F-FDG is primarily employed to trace the utilization of glucose within the brain and associated PD-related metabolic changes. In this study, ^18^F-FDG PET/CT imaging was conducted to assess cerebral glucose metabolism in mice with MPTP-induced PD on day 7. Relative to control animals, those in the treated MPTP group exhibited significantly decreased ^18^F-FDG uptake, consistent with metabolic abnormalities (Figure 5(c)). However, such energy metabolism was clearly restored to levels close to those in control animals following MAG-NCs@Gel treatment. The energy metabolism levels in these mice were improved to some degree in the MAG and MAG-NCs groups as well, although the effects were not as robust as those in the MAG-NCs@Gel group (Figure S15). When further assessing changes in cerebellar glucose metabolism (MRglc), the cerebellar MRglc levels of mice in the MAG-NCs@Gel group remained at 2.058 ± 0.171 and were close to the levels in control animals and higher than levels in animals in any other experimental treatment groups (Figure 5(d)). Changes in body weight over time following drug treatment can additionally reflect the restoration of normal MRglc. Relative to the steady weight gain observed in the control group, mice in the L-DOPA or MAG group initially exhibited some degree of weight loss followed by gradual weight increases, consistent with MRglc recovery (Figure S16). To establish the protective effects of this MAG-NCs@Gel sustained-release platform on mitochondrial function within the midbrain MAG-NCs platform on midbrain mitochondria, MPTP-induced PD model animals were again utilized. In this model system, MPTP is converted into toxic MPP^+^ within dopaminergic neurons, wherein it agglomerates within the mitochondrial and blocks complex I of the electron transport chain. Mitochondrial function in treated animals was assessed by measuring ATP, ROS, and malondialdehyde (MDA) levels. As demonstrated in Figure 5(e), the level of ATP in the MPTP group (33.54 ± 2.98 ng mL^−1^) decreased significantly relative to the control group (53.64 ± 4.33 ng mL^−1^). Relative to the MPTP group, the ATP levels in the MAG-NCs@Gel group (52.13 ± 4.82 ng mL^−1^) significantly increased, approaching levels in the control and L-DOPA groups (Figure S17a). ROS levels in MPTP model animals were roughly twofold higher than those in control animals (Figure 5(f)), while levels of the oxidative stress indicator MDA were similarly increased in the MPTP group (Figure 5(g)). Following MAG-NCs@Gel treatment, ROS and MDA levels returned to normal, consistent with the excellent therapeutic efficacy of this therapeutic platform within this murine model system owing to its ability to efficiently reverse MPTP-induced mitochondrial dysfunction. MDA and ROS levels were also decreased in other treatment groups, but these effects were not as pronounced as in the MAG-NCs@Gel group (Figure S17b and c). The mechanism whereby this MAG-NCs@Gel release platform can treat mitochondrial dysfunction is summarized in Figure 5(h). Initially, MAG-NCs are continuously released from the PNIPAM hydrogel and can readily pass through the BBB whereupon they enter dopaminergic neurons via endocytosis to regulate mitochondrial ATP, ROS, and MDA levels so that they return to normal, thereby abrogating MPTP-induced neurotoxicity and facilitating neuroprotection. The behavioral skills of animals in these studies improved significantly to near baseline levels, thus confirming the effective treatment of PD in these animals.

### 2.6. In Vivo Biocompatibility Analysis

**Figure 6:**
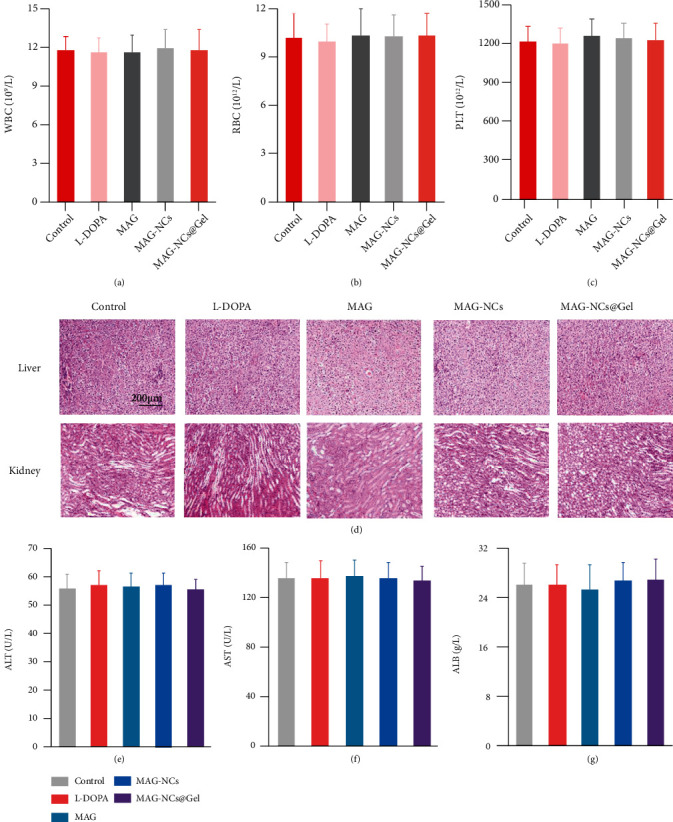
Biocompatibility analysis. Assessment of in vivo biocompatibility in different treatment groups. Routine blood analyses of (a) WBC, (b) RBC, and (c) (PLT). (d) Representative H&E-stained liver and kidney tissue sections. Liver and kidney functional parameters were measured, including (e) ALT, (f) AST, and (g) ALB.

Following a 7-day treatment period, MAG-NCs@Gel biocompatibility following intranasal administration was assessed by examining nasal mucosal sections, performing routine blood analyses, and assessing organ inflammation or lesions. Relative to samples from the control group, no evidence of inflammation or structural damage was observed in samples from animals treated with blank hydrogels or different MAG treatments (Figure S18). Additionally, no differences in routine blood analysis parameters (WBC, RBC, and PLT) were observed in the MAG treatment groups relative to the control or L-DOPA treatment groups (Figures 6(a)–6(c)). Consistently, routine blood parameters (HGB, MCV, MCH, MCHC, and HCT) were also normal in these animals (Figure S19). H&E-stained sections of major tissues including the liver and kidneys exhibited no evidence of inflammation or injury in any treatment group (Figure 6(d)), with no evidence of inflammation in the heart, lung, brain, or spleen sections from animals in the MAG-NCs@Gel group or other treatment groups (Figure S20). Given the synthetic nature of the PNIAM hydrogel, there is an associated risk of organ rejection or dysfunction, but no such abnormalities were observed when assessing liver and kidney function in treated mice (Figures 6(e)–6(g) and Figure S21). Therefore, these data suggested that our MAG-NCs@Gel sustained-release system exhibited satisfactory biocompatibility following intranasal injection, making it of potential value for the treatment of PD.

## 3. Discussion

In summary, this study developed a thermosensitive PNIPAM hydrogel for encapsulating MAG-NCs to facilitate sustained drug delivery following intranasal administration, thereby effectively treating a murine model of PD owing to associated self-gelation processes. It is needed to point out that the presence of MAG-NCs enhances the stability and strength of PNIPAM hydrogel, indicating the synergic action of weak interactions among MAG-NCs and PNIPAM chains. The LCST behavior of the drug delivery platform prolonged the residence time of MAG-NCs in the nasal cavity so that these MAG-NCs were able to readily cross the BBB to achieve the targeted regulation of ROS and ATP levels in dopaminergic neurons in the context of MPTP-induced mitochondrial dysfunction. MAG-NCs@Gel treatment was associated with significant reductions in dopaminergic neuron neurotoxicity in PD model mice, with concomitant improvements in neuroprotection and motor behavior. Importantly, this drug delivery platform also exhibited satisfactory biocompatibility without any evident side effects.

Together, these results underscore the potential value of intranasal MAG-NCs@Gel injection for the treatment of PD, overcoming key limitations to traditional drug delivery such as the rapid loss of drugs from the nasal cavity and the restricted capability of drugs to cross the BBB. In addition, the work may realize controlled transmembrane of drug NCs and targeted treatment for Parkinson's disease, Alzheimer's disease, and other neurodegenerative diseases.

## Data Availability

All data needed of this study are available in the article and its Supplementary Information files.

## Supplementary Materials

Supplementary 1 Materials and Methods: reagents, preparation of MAG-NCs, preparation of MAG-NCs@Gel, characterizations, and analysis of hydrogel viscoelasticity. Cell and animal experiments: ex vivo nasal mucosa permeation, cytotoxicity assessment, analysis of in vitro neuroprotective effect, flow cytometry, pharmacokinetics, therapeutic effects on MPTP-induced PD mouse model, behavioral evaluations, PET/CT imaging, immunofluorescence staining, determination of DA level in striatum, evaluation of mitochondrial function in midbrain, and in vivo biocompatibility analysis. Figure S1: (a) dependence transparency of hydrogels on temperature. (b) Estimating the LCST of hydrogels through differentiation of transparency to temperature. Figure S2: the temperature dependence of MAG-NCs@Gel viscoelasticity. Figure S3: particle size distribution (a) and TEM image (b) of MAG-NCs, (c) particle size of MAG-NCs for different storage times, and (d) TEM image of MAG-NCs following storage for 14 days at 4oC. Figure S4: SEM images of freeze-dried blank hydrogel. Figure S5: SEM images of freeze-dried MAG-NCs@Gel following storage for 14 days at room temperature. Figure S6: FT-IR of MAG-NCs@Gel. Figure S7: rheological properties of hydrogels as a function of oscillation strain. Figure S8: evaluation of injectability based on changes of *G*′ and *G*^″^ of MAG-NCs@Gel under low (1%) and high (200%) oscillatory shear strain. Figure S9: the dependence of *G*′ and *G*^″^ of blank hydrogel on angular frequency. Figure S10: ex vivo nasal mucosa permeation curve for MAG-NCs as a function of time. Figure S11: cell viability following incubation with different concentrations of treatment groups. Figure S12: drug-mediated protection against MPP^+^-induced cell death and cytotoxicity. ^#^*p* < 0.05 and ^##^*p* < 0.01 vs. the MAG group. Figure S13: MAG-NCs and MAG-NCs@Gel pharmacokinetics in mice. (a) Plasma and (b) brain concentration-time profiles (*n* = 6 and 4, respectively). Figure S14: striatum parameters (c) DA, (d) DOPAC, and (e) HVA. ^∗^*p* < 0.05 and ^∗∗^*p* < 0.01 vs. the MPTP group, ^#^*p* < 0.05 and ^##^*p* < 0.01 vs. the MAG group, ^▲^*p* < 0.05 and ^▲▲^*p* < 0.01 vs. the MAG-NCs group. Figure S15: ^18^F-FDG PET images of brain anatomy in the coronal (left), sagittal (middle), and transverse directions (right). (a) MAG group. (b) MAG-NCs group. Figure S16: body weight tracking for MPTP-inducted PD mice in the indicated treatment groups over a 7-day period. Figure S17: mitochondrial function parameters for different treatment groups were detected in the midbrain, (a) ATP level, (b) MDA level, and (c) ROS level. ^#^*p* < 0.05 and ^##^*p* < 0.01 vs. the MAG group. Figure S18: nasal mucosal sections from different groups. Figure S19: routine blood analysis parameters. Figure S20: representative images of H&E stained organs. Figure S21: quantitative assessment of liver and kidney function parameters. Table S1: plasma and brain pharmacokinetic criteria associated with MAG-NCs and MAG-NCs@Gel. Table S2: plasma and brain pharmacokinetic criteria associated with intravenous administration (MAG-NCs) and intranasal administration (MAG-NCs@Gel) (*n* = 6 or 4).

Supplementary 2 Movie S1: self-gelation process of MAG-NCs@Gel.

Supplementary 3 Movie S2: demonstration of injectable properties of MAG-NCs@Gel.
