# Supplementary material for: Rational Design of Thermosensitive Hydrogel to Deliver Nanocrystals with Intranasal Administration for Brain Targeting in Parkinson's Disease
Source: Research (Wash D C). 2021 Nov 19;2021:9812523. doi: 10.34133/2021/9812523 (PMC8627567; doi:10.34133/2021/9812523)
Supplement: Supplementary 1 — Materials and Methods: reagents, preparation of MAG-NCs, preparation of MAG-NCs@Gel, characterizations, and analysis of hydrogel viscoelasticity. Cell and animal experiments: ex vivo nasal mucosa permeation, cytotoxicity assessment, analysis of in vitro neuroprotective effect, flow cytometry, pharmacokinetics, therapeutic effects on MPTP-induced PD mouse model, behavioral evaluations, PET/CT imaging, immunofluorescence staining, determination of DA level in striatum, evaluation of mitochondrial function in midbrain, and in vivo biocompatibility analysis. Figure S1: (a) dependence transparency of hydrogels on temperature. (b) Estimating the LCST of hydrogels through differentiation of transparency to temperature. Figure S2: the temperature dependence of MAG-NCs@Gel viscoelasticity. Figure S3: particle size distribution (a) and TEM image (b) of MAG-NCs, (c) particle size of MAG-NCs for different storage times, and (d) TEM image of MAG-NCs following storage for 14 days at 4oC. Figure S4: SEM images of freeze-dried blank hydrogel. Figure S5: SEM images of freeze-dried MAG-NCs@Gel following storage for 14 days at room temperature. Figure S6: FT-IR of MAG-NCs@Gel. Figure S7: rheological properties of hydrogels as a function of oscillation strain. Figure S8: evaluation of injectability based on changes of G′ and G″ of MAG-NCs@Gel under low (1%) and high (200%) oscillatory shear strain. Figure S9: the dependence of G′ and G″ of blank hydrogel on angular frequency. Figure S10: ex vivo nasal mucosa permeation curve for MAG-NCs as a function of time. Figure S11: cell viability following incubation with different concentrations of treatment groups. Figure S12: drug-mediated protection against MPP+-induced cell death and cytotoxicity. #p < 0.05 and ##p < 0.01 vs. the MAG group. Figure S13: MAG-NCs and MAG-NCs@Gel pharmacokinetics in mice. (a) Plasma and (b) brain concentration-time profiles (n = 6 and 4, respectively). Figure S14: striatum parameters (c) DA, (d) DOPAC, and (e) H [file 9812523.f1.docx]

Supplementary Information

**Rational Design of Thermosensitive Hydrogel to Deliver Nanocrystals with Intranasal Administration for Brain Targeting in Parkinson's Disease**

Yun Tan,^1^† Yao Liu,^2^† Yujing Liu,^2^ Rui Ma,^2^ Jingshan Luo,^2^ Huijie Hong,^3^ Xiaojia Chen,^3^ Shengpeng Wang,^3^ Chuntai Liu,^4^ Yi Zhang,^1^* and Tongkai Chen^2^*

1 Hunan Provincial Key Laboratory of Micro & Nano Materials Interface Science, College of Chemistry and Chemical Engineering, Central South University, Changsha 410083, China.

2 Science and Technology Innovation Center, Guangzhou University of Chinese Medicine, Guangzhou 510405, China

3 State Key Laboratory of Quality Research in Chinese Medicine, Institute of Chinese Medical Sciences, University of Macau, Macau 999078, China

4 Key Laboratory of Materials Processing and Mold, Ministry of Education, Zhengzhou University, Zhengzhou, 450002, China

* Correspondence should be addressed to Yi Zhang; yzhangcsu@csu.edu.cn and Tongkai Chen; [chentongkai@gzucm.edu.cn](mailto:chentongkai@gzucm.edu.cn)

† Yun Tan and Yao Liu contributed equally to this work.

1. **Materials and Methods**

***1.1. Reagents.*** *N*-isopropylacrylamide (NIPAM), gelatin methacryloyl (GelMA), potassium peroxydisulfate (KPS), and tetramethylethylenediamine (TEMED) were purchased from Aladdin Co. Ltd. 1-methyl-4-phenylpyridinium ion (MPP^+^), 1-methyl-4-phenyl-1,2,3,6-tetrahydropyridine (MPTP), rabbit polyclonal anti-TH, and Levodopa (L-DOPA) were obtained from Sigma-Aldrich (St. Louis, MO, USA). Deionized water (18.2 MΩ at 25^o^C) was provided for all experiments using a water pool ultrapure water purification system (WP-UP-YJ-20).

***1.2. Preparation of MAG-NCs.*** An antisolvent precipitation approach was employed for MAG-NC preparation. In brief, the injection of 20 mg/mL of MAG in 0.5 mL of acetone was done into 25 mL of water containing 0.6 mg/mL of polyvinylpyrrolidone K30 (PVP-K30) as a stabilizer, with the mixture being constantly stirred at room temperature at 1000 rpm

***1.3. Preparation of MAG-NCs@Gel.*** *N*-isopropylacrylamide (1.02 g) was dissolved in 3 mL deionized water, and a 1.5 mL volume of 0.5 wt% gelatin methacryloyl was added, following which the mixture was stirred for 10 min. Next, 1 mL of 2 wt% of KPS solution and TEMED were added to the ice water bath and stirred for 5 min to yield a uniform precursor preparation that was stored for 6 h at 60^o^C to prepare an emulsion. This emulsion was then combined with 5 mL of the prepared MAG-NCs solution, with the mixture then being stirred for 5 min at 60^o^C. The resultant MAG-NCs@Gel preparation was then obtained by natural cooling of the stationary solution. The MAG-NC-free blank PNIPAM hydrogel was also prepared.

***1.4. Characterizations.*** MAG-NCs particle scale and dispersion were ascertained via transmission electron microscopy (TEM) and dynamic light scattering (DLS). For DLS analyses, particle hydrodynamic sizes were assessed with a Malvern Zetasizer Nano S (Malvern Instruments Ltd., UK). TEM images of MAG-NCs and MAG-NCs@Gel were captured with a TEM (Tecnai G2 Spirit TWIN, FEI) at an accelerating voltage of 80.0 kV. Freeze-dried MAG-NCs@Gel and Gel microstructural analyses were performed by utilizing a scanning electron microscope (SEM, JSM-7900F, JEOL) at an accelerating voltage of 20 kV. Fourier transform infrared spectroscopy (FT-IR) was executed with a PerkinElmer (L16000300 Spectrum TWO LITA, Llantrisant, UK) from 4000 to 500 cm^-1^ through the approach of potassium bromide pellet.

***1.5. Analysis of Hydrogels Viscoelasticity.*** An Anton Paar rheometer (MCR 92) was used to assess hydrogel viscoelasticity for parallel platelets of 15 mm in diameter. An amplitude sweep was performed for the 0.1%-100% strain range, with an oscillation frequency of 6.28 rad s^-1^ at 25^o^C, while the frequency sweep was executed in the 0.1-100 rad s-1 range at a fixed strain of 1% at 25^o^C. Temperature sweep conducted from 25^o^C – 40^o^C with a fixed strain of 1% and an oscillation frequency of 6.28 rad s^-1^. To assess shear viscosity, the rate of shear was enhanced from 0.1 to 100 s^−1^ and then reduced from 100 s^-1^ to 0.1 s^-1^ at 25°C. Time sweeps for small and large oscillatory strains were switched from γ = 1% and γ = 200%, respectively, with a strain interval of 120 s.

***1.6. Ex Vivo Nasal Mucosa Permeation.*** MAG permeations were assessed using Franz diffusion cells. Briefly, samples of porcine nasal mucosa were fixed between the donor and recipient compartments so that the front and back of the mucosa were facing the receiver and donor compartments, respectively. Both compartments were filled with 6 mL of phosphate buffer (pH 6.8) complemented with 0.5% (w/v) sodium dodecyl sulfate (SDS). The recipient compartment was continuously stirred at 200 rpm and was warmed to 34 ± 0.5℃. At specific time points (0, 0.25, 0.5, 1, 2, 4, 8, 12, 24, 48, and 72 h), 0.5 mL of media was eliminated and substituted with an equivalent of fresh media. MAG levels were assessed via high-performance liquid chromatography (HPLC) applying a quaternary pump and a reversed-phase C18 column (250 × 4.6 mm × 5 μm). Temperatures were maintained at 30℃ and detection was conducted at 290 nm. A methanol-water mixture (75:25, v/v) was utilized as a mobile-phase, with a rate of flow of about 1.0 mL/min and a volume of injection of about 10 μL.

***1.7. Cytotoxicity Assessment.*** Human SH-SY5Y neuroblastoma cells were cultured within DMEM comprising 10% fetal bovine serum (FBS) and 1% penicillin/streptomycin in a 5% CO_2_ incubator at 37°C. To assess the cytotoxicity, SH-SY5Y cells were added to the plates containing 96 wells (5000/well) and cultured for 24 h, after which they were processed with a range of MAG or MAG-NCs concentrations (5, 10, 20, 40, 80, and 160 μM) for an additional 24 h. 10 μL MTT was added and the incubation of plates was performed for 4 h. Further, 100 μl DMSO was introduced and the absorbance was evaluated at 490 nm.

***1.8. Analysis of in Vitro Neuroprotective Effect****.* SH-SY5Y cells were added to the plates containing 96 wells (5×103/well) for 24 h, after which they were treated for 2 h with MAG or MAG-NCs (30 μM). Next, MPP^+^ was increased to each well (2 mM) and the incubation of cells was accomplished for 36 h, after which an MTT assay was performed as above. In addition, cells treated with 30 μM MAG preparations were stained with calcein-AM/PI and assessed via confocal microscopy to detect live and dead cells.

***1.9. Flow Cytometry.*** SH-SY5Y cells were added to the plates containing 6 wells (3 × 105/well) and grown to 80% confluence, after which they were treated for 2 h with MAG or MAG-NCs (30 μM). Next, MPP^+^ was increased to each well (2 mM) and the incubation of cells was accomplished for 36 h. Subsequently, cells were collected, stained by utilizing 5 μL of Annexin V-FITC and 10 μL of propidium iodide (PI), and rates of apoptosis were assessed via flow cytometry.

***1.10. Pharmacokinetics.*** The Animal Ethics Committee of Guangzhou University of Chinese Medicine confirmed all animal investigations explained in this research, which were performed in compliance with national regulatory principles. Male Sprague-Dawley (SD) rats (6-8 weeks old) from Guangdong Medical Laboratory Animal Centre (Guangdong, China) were housed under standard laboratory circumstances (25 ± 2°C; 12 h dark/light cycle; 55 ± 5% relative humidity) with unrestricted accessibility of water and food. Animals were separated into an intravenous (IV) group and an intranasal (IN) administration group at random, with 6 rats per group for a plasma pharmacokinetic study.(1) MAG, MAG-NCs@Gel, and MAG-NCs (dose 1.0 mg/kg) were administered intranasally or intravenously to each group, respectively, with samples of blood (300 μL per time point) being collected from the posterior orbital venous plexus at appropriate time points (before administration and at different 0.083, 0.25, 0.5, 1, 2, 3, 4, 6, 8, 12, 24, 48 h post-administration). After collection, the samples of blood were spun at 5,000 rpm for 10 min, and plasma was collected from these samples, with acetone being used to determine the MAG concentrations therein.

For brain pharmacokinetic studies, four rats were respectively assigned to the IV and the IN groups. Following administration of the appropriate MAG preparations, rats were euthanized via the cervical dislocation, after which, cardiac perfusion with 30 mL of normal saline was performed. Brain samples were dissected from these animals, washed twice with normal saline, and all adherent tissues and fluids were removed.

Plasma and brain MAG concentrations were ascertained via LC-MS/MS.(2) The time to attain the maximum concentration (T_max_), the maximum concentration (C_max_), and the area under the curve (AUC) for MAG in the plasma and brain were additionally measured, and pharmacokinetic profiles for MAG were established through plotting drug concentration vs time curves. In addition, the MAG-NCs@Gel direct transport percentage (DTP %) and targeting efficiency (DTE %) were calculated by utilizing the following equation (1) and (2):

$\text{DTE \%=}\frac{{\text{[}\text{AUC}_{\text{Brain}}\text{/}\text{AUC}_{\text{Blood}}\text{]}}^{\text{(IN)}}}{{\text{[}\text{AUC}_{\text{Brain}}\text{/}\text{AUC}_{\text{Blood}}\text{]}}^{\text{(IV)}}}\text{×100}$ (1)

$\text{DTP \%=}\frac{\text{(B)}_{\text{IN}}\text{-(}\text{B}_{\text{X}}\text{)}}{\text{(B)}_{\text{IN}}}\text{×100}$ (2)

In these equations, Bx =(BIV/PIV) ×PIN, Bx represents the brain AUC fraction contributed through systemic circulation via the BBB after intranasal administration, BIV implies the AUC0–8 h (brain) after intravenous administration, PIV denotes the AUC0–8 h (blood) after intravenous administration, BIN is the AUC0–8 h (brain) after intranasal administration and PIN implies the AUC0–8 h (blood) after intranasal administration.

***1.11. Therapeutic Effects on MPTP-induced PD Mouse Model****.* Male C57BL/6 mice were randomized into the following groups: (1) control, (2) MPTP, (3) L-DOPA, (4) MAG, (5) MAG-NCs, and (6) MAG-NCs@Gel. PD model mice were established through the intraperitoneal injection of MPTP (18 mg/kg) every 2 h, with four total injections per mouse. Control mice were administered equivalent amounts of 0.9% saline solution. During the next 7 days, mice were given appropriate treatments once per day (Group 1-2: 0.9% saline; Group 3: L-DOPA dose = 25 mg/kg; Group 4-6: MAG equivalent dose = 1.0 mg/kg via intranasal administration).

***1.12. Behavioral Evaluations.*** Behavioral testing was performed for mice in Groups 1-6 on day 7 post-treatment. In pole tests, mice were placed atop a vertical pole (diameter = 1 cm, height = 50 cm) with a rough surface. The time that was taken by the mice to turn their head downward (T-turn) and the time to attain the bottom (T-total) were recorded. For rotarod tests, mice were located atop the rod in a separate compartment and analyzed for 20 min with a rotation speed of 20 rpm. The fall latency and total drops per mouse were recorded. Both of tests were conducted thrice. In tests of locomotor activity, mice were added to an activity monitoring chamber (60 cm × 60 cm × 40 cm), and movements were recorded for 20 min, with speed being calculated with the Open field computer program (Clever Sys Inc., VA, USA).

***1.13. PET/CT Imaging.*** PET/CT imaging conducted on day 8 in order to evaluate brain energy metabolism. Mice were fasted for 12 h, prior to the PET imaging, following which they were intravenously administered 200 ± 10 μC_i_ of [18]-fluoro-6-deoxy-glucose ([18F]-FDG). At 1 h post-[18F]-FDG uptake, mice were anesthetized by utilizing 2% isoflurane and located on a scanning bed.(3) The images of PET/CT were then captured in static mode for 10 min, after which a CT scan was performed in normal mode with a TransPET Discoverist 180 system (Raycan Technology Co., Ltd, Suzhou, China). A three-dimensional (3D) OSEM method was used for PET image reconstruction with a voxel scale of 0.5×0.5×0.5 mm^3^, while the images of CT were reconstructed with an FDK algorithm and a 256 × 256 × 256 matrix. The AMIDE (Amide's a Medical Imaging Data Examiner) and Pmod (Pmod Technologies LLC, Switzerland) computer programs were used for image display. The mean standardized uptake value (SUV) was measured as given below: mean pixel value with the decay-corrected region-of-interest activity (μC_i_/kg)/ (injected dose [μC_i_]/weight [kg]).

***1.14. Immunofluorescence Staining.*** Animals were euthanized, and tissue samples were collected to conduct immunofluorescent staining in order to quantify the numbers of TH-positive neurons within the substantia nigra pars compacta (SNpc). Briefly, 30 μm coronal sections were collected from AP −2.80 mm to AP −3.97 mm. Rabbit anti-mouse TH (Abcam, 1:1000) served as a primary antibody, while Alexa Fluor 594-conjugated anti-rabbit IgG (Cell Signaling Technology, 1:1000) served as a secondary antibody. A fluorescence microscope (Model DMi8, Leica, Germany) was used to image stained cells, with ImageJ being used to quantify numbers of TH-positive cells.

***1.15. Determination of DA Level in Striatum.*** Samples were isolated and prepared as in prior reports.(4) Briefly, concentrations of DA and its metabolites 3,4-dihydroxyphenylacetic acid (DOPAC) and homovanillic acid (HVA) were assessed via chromatography (ESA, MA, USA) using a 5014B electrochemical detector.

***1.16. Evaluation of Mitochondrial Function in Midbrain****.* Tissues were rapidly removed, homogenized in ice-cold 0.9% saline, and then centrifuged at 1,000×g at 4°C for 10 min. Supernatants were then transferred to a tube for subsequent analysis, with ATP, ROS, and malondialdehyde (MDA) levels therein being assessed using goat anti-mouse ATP, ROS, and MDA ELISA kits, respectively, based upon provided directions.

***1.17. In Vivo Biocompatibility Analysis.*** Mice were euthanized, and samples of the nasal septum with the intact epithelial cell membrane on each side were carefully separated from the bone, fixed, prepared into sections, stained by implementing eosin (H&E) and hematoxylin, and appraised via light microscopy. Murine blood was collected for analysis. The collection of major organs (liver, lungs, kidneys, heart, spleen) was carried out, fixed with 4% paraformaldehyde, paraffin-embedded, cut into 4 μm-thick sections, and subjected to H&E staining for pathological analyses.

**2. Figures**


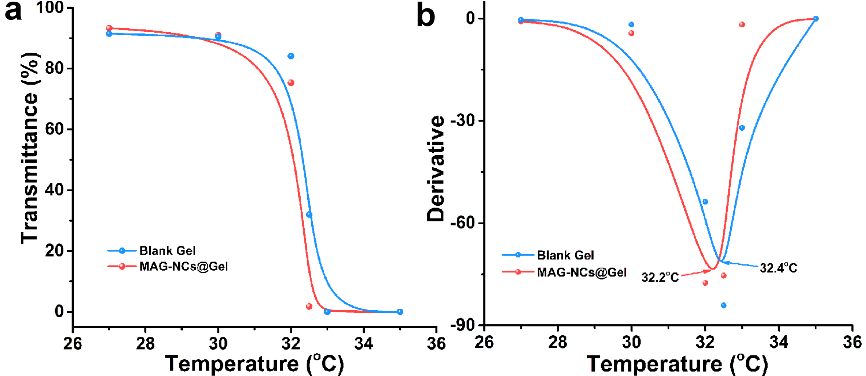


Figure S1. (a) Dependence transparency of hydrogels on temperature. (b) Estimating the LCST of hydrogels through differentiation of transparency to temperature.





Figure S2. The temperature dependence of MAG-NCs@Gel viscoelasticity.


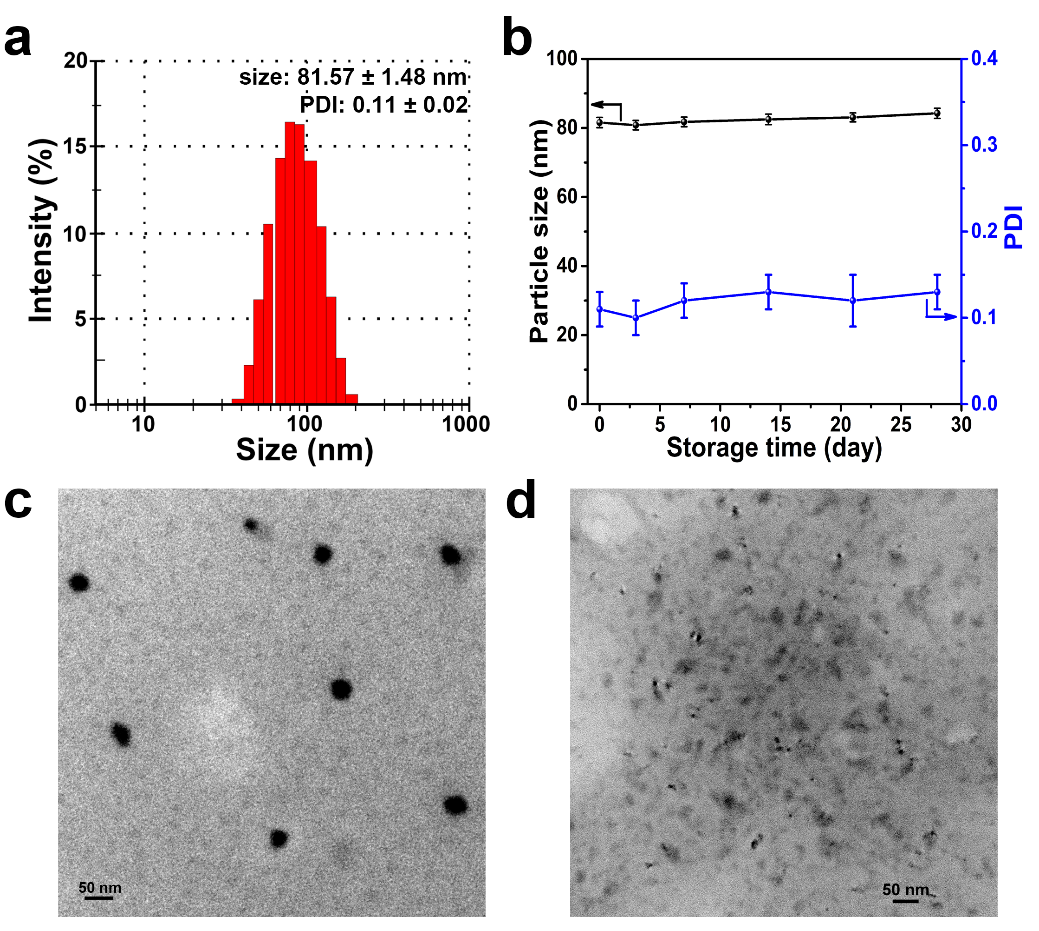


Figure S3 Particle size distribution (a) and TEM image (b) of MAG-NCs, (c) Particle size of MAG-NCs for different storage times, (d) TEM image of MAG-NCs following storage for 14 days at 4^o^C.


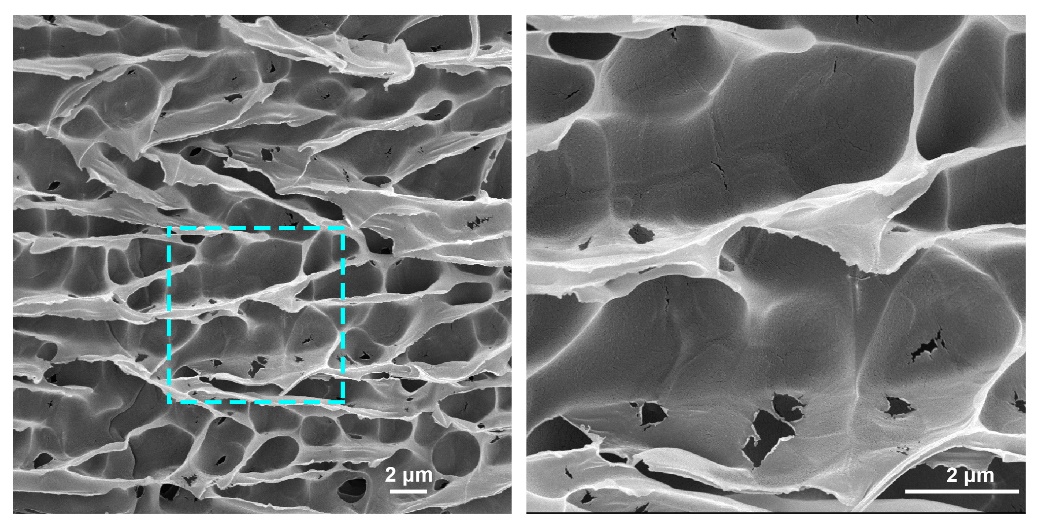


Figure S4. SEM images of freeze-dried blank hydrogel.


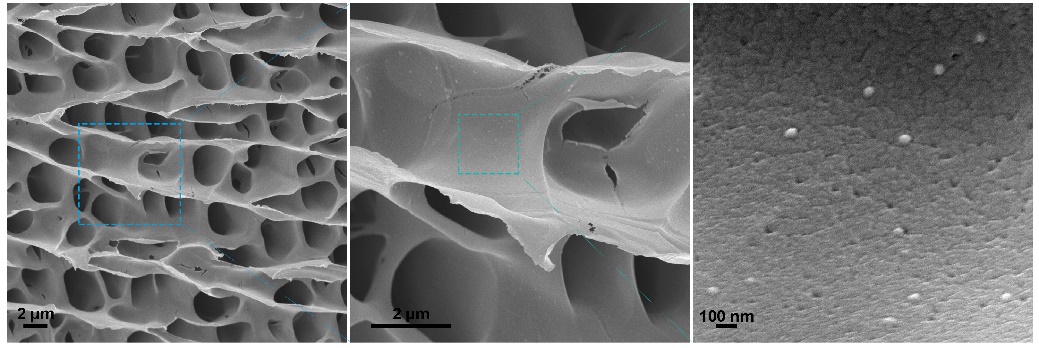


Figure S5. SEM images of freeze-dried MAG-NCs@Gel following storage for 14 days at room temperature.





Figure S6. FT-IR of MAG-NCs@Gel.





Figure S7. Rheological properties of hydrogels as a function of oscillation strain.


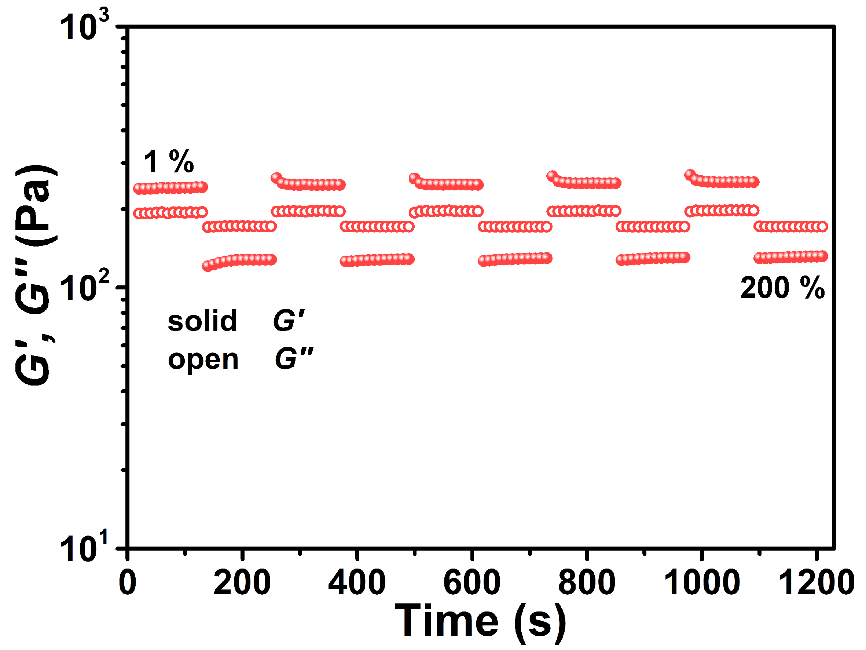


Figure S8. Evaluation of injectability based on changes of G' and G'' of MAG-NCs@Gel under low (1%) and high (200%) oscillatory shear strain.





Figure S9. The dependence of G' and G'' of blank hydrogel on angular frequency.





Figure S10. *Ex vivo* nasal mucosa permeation curve for MAG-NCs as a function of time.





Figure S11. Cell viability following incubation with different concentrations of treatment groups.





Figure S12. Drug-mediated protection against MPP^+^-induced cell death and cytotoxicity. ^#^p<0.05 and ^##^p<0.01 vs MAG group.


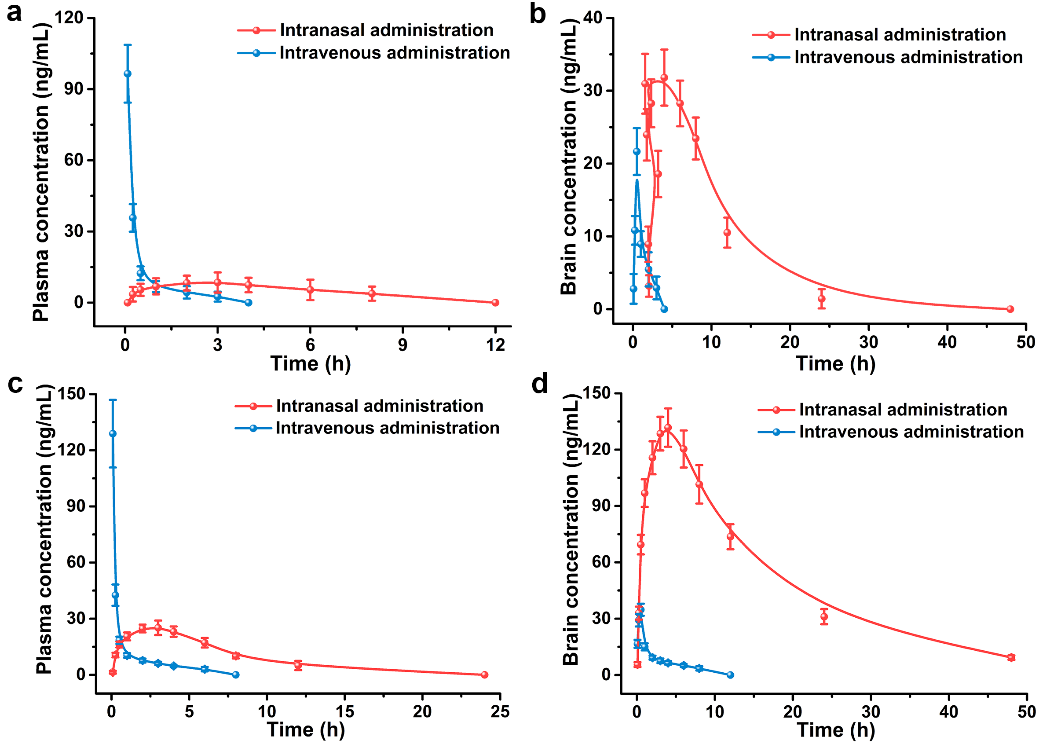


Figure S13. The pharmacokinetics of MAG alone via intravenous (a) and intranasal administration (b); MAG-NCs and MAG-NCs@Gel pharmacokinetics in mice. pharmacokinetics of MAG with Plasma (c) and brain (d) concentration−time profiles (n = 6 and 4, respectively).


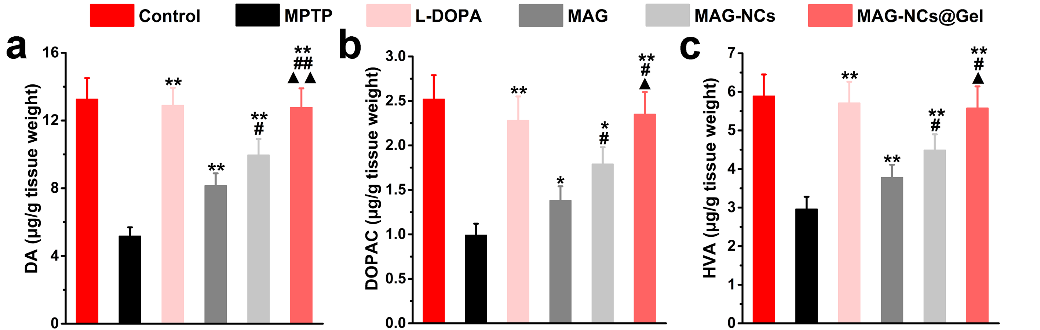


Figure S14. Striatum parameters (c) DA, (d) DOPAC and (e) HVA. *p<0.05 and **p<0.01 vs MPTP group, #p<0.05 and ##p<0.01 vs MAG group, ▲p<0.05 and ▲▲p<0.01 vs MAG-NCs group.


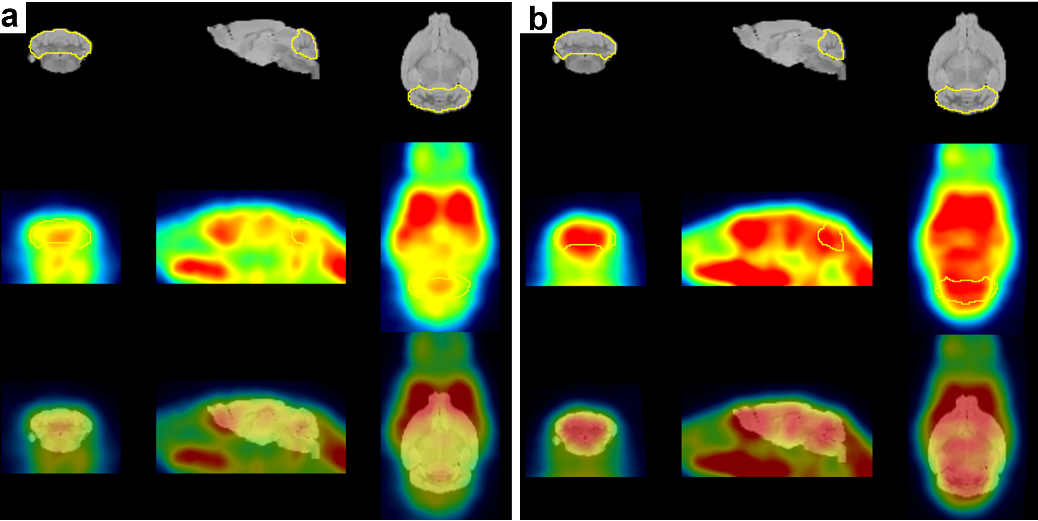


Figure S15. ^18^F-FDG PET images of brain anatomy in the coronal (left), sagittal (middle), and transverse directions (right). (a) MAG group, (b) MAG-NCs group.





Figure S16. Body weight tracking for MPTP-inducted PD mice in the indicated treatment groups over a 7-day period.


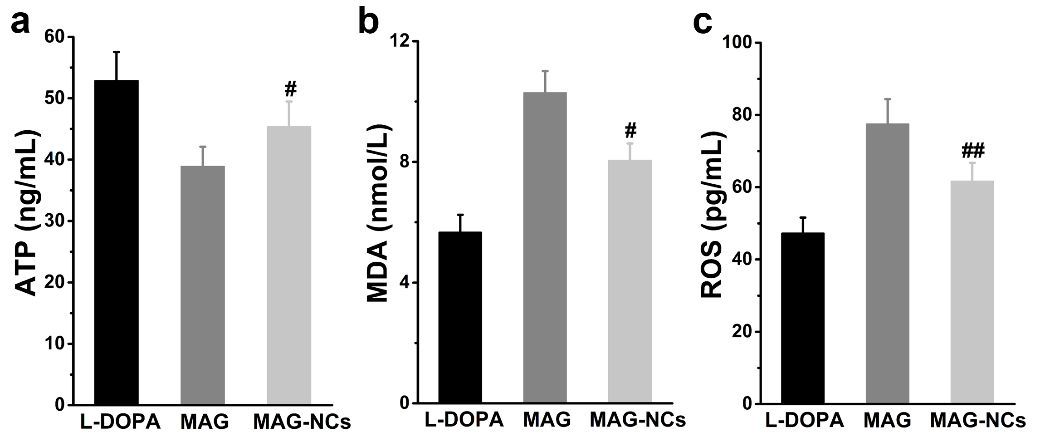


Figure S17. Mitochondrial function parameters for different treatment groups were detected in the midbrain, (a) ATP level, (b) MDA level and (c) ROS level. #p<0.05 and ##p<0.01 vs MAG group.


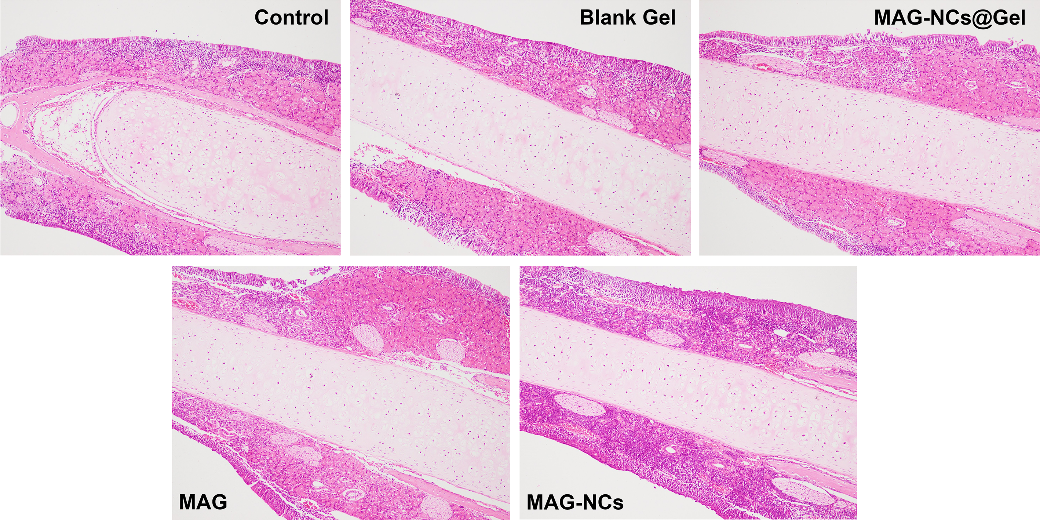


Figure S18. Nasal mucosal sections from different groups.


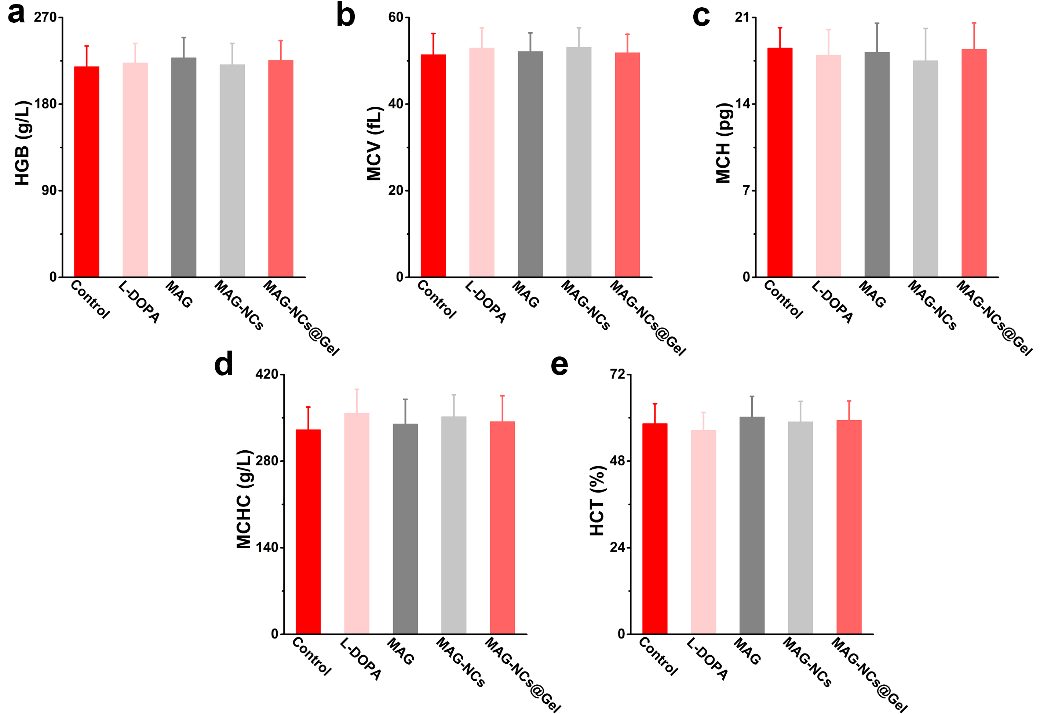


Figure S19. Routine blood analysis parameters.


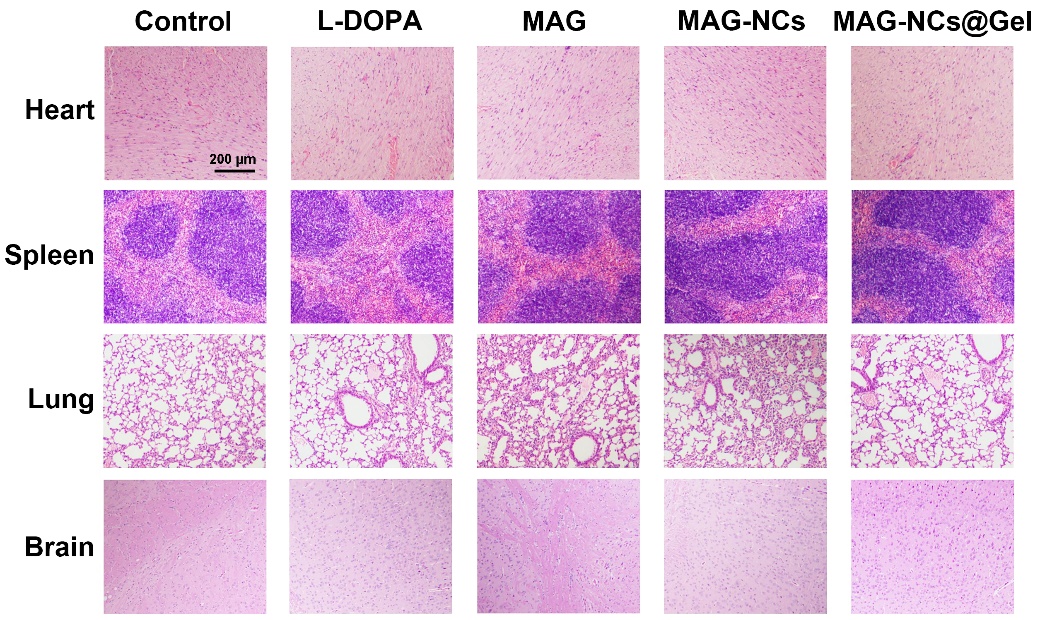


Figure S20. Representative images of H&E stained organs.


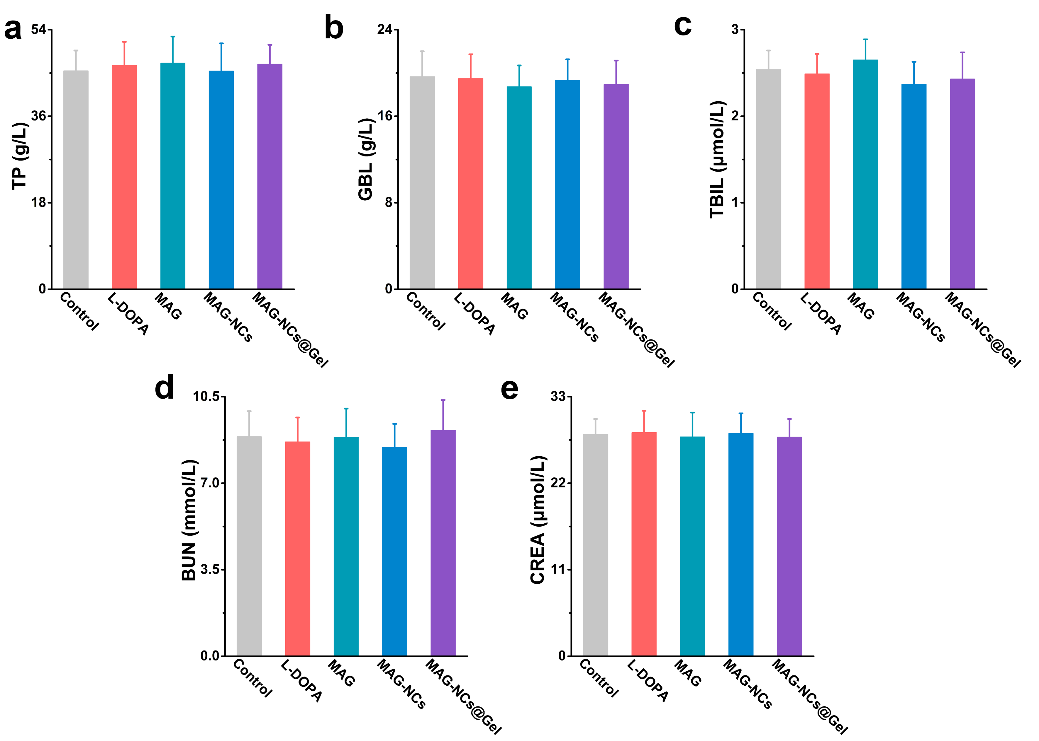


Figure S21. Quantitative assessment of liver and kidney function parameters.

**3. Table**

Table S1. Plasma and brain pharmacokinetic parameters after intravenous administration and intranasal administration of MAG (n = 4) *^a^*

| Parameters | Intravenous administration | Intranasal administration |
| --- | --- | --- |
| Plasma | | |
| T_1/2_ (h) | 1.42 ± 0.20 | 4.15 ± 0.53^*^ |
| T_max_ (h) | 0.083 ± 0 | 3.25 ± 0.50 |
| C_max_ (ng/ml) | 97.79 ± 10.24 | 9.03 ± 0.98^*^ |
| AUC_0-t_ (ng.h/ml) | 40.43 ± 4.17 | 50.97 ± 6.09^**^ |
| MRT_0-t_ (h) | 0.56 ± 0.15 | 3.69 ± 0.51^**^ |
| Brain | | |
| T_1/2_ (h) | 1.23 ± 0.22 | 4.18 ± 0.65^*^ |
| T_max_ (h) | 0.5 ± 0 | 4.50 ± 1.00 |
| C_max_ (ng/g) | 22.85 ± 3.94 | 33.01 ± 4.03^*^ |
| AUC_0-t_ (ng.h/g) | 24.46 ± 2.09 | 353.87 ± 37.38^**^ |
| MRT_0-t_ (h) | 1.09 ± 0.17 | 7.01± 0.88^**^ |
| Brain/plasma ratio | | |
| DTE | 706.17% | |
| DTP | 81.28% | |

*^a^* *p<0.05 and **p<0.01 vs the intravenous group.

Table S2. Plasma and brain pharmacokinetic criteria associated with intravenous administration (MAG-NCs) and intranasal administration (MAG-NCs@Gel) (n = 6 or 4) *^a^*

| Parameters | Intravenous administration | Intranasal administration |
| --- | --- | --- |
| Plasma | | |
| T_1/2_ (h) | 1.44 ± 0.17 | 3.05 ± 0.41^*^ |
| T_max_ (h) | 0.083 ± 0 | 3.50 ± 0.55^**^ |
| C_max_ (ng/ml) | 128.87 ± 18.06 | 25.13 ± 3.87^**^ |
| AUC_0-t_ (ng.h/ml) | 89.27 ± 10.23 | 302.07 ± 29.86^**^ |
| MRT_0-t_ (h) | 1.70 ± 0.22 | 5.68 ± 0.47^*^ |
| Brain | | |
| T_1/2_ (h) | 3.03 ± 0.35 | 11.51 ± 1.42^**^ |
| T_max_ (h) | 0.63 ± 0.31 | 4.67 ± 1.03^**^ |
| C_max_ (ng/g) | 34.76 ± 3.19 | 131.75 ± 10.21^**^ |
| AUC_0-t_ (ng.h/g) | 93.24 ± 10.96 | 2555.69 ± 336.17^**^ |
| MRT_0-t_ (h) | 3.07 ± 0.24 | 13.27 ± 1.08^**^ |
| Brain/plasma ratio | | |
| DTE | 809.98% | |
| DTP | 87.65% | |

*^a^* *p<0.05 and **p<0.01 vs the intravenous group.

**References**

1. T. K. Chen, W. Liu, S. Xiong, D. L. Li, S. H. Fang, Z. F. Wu, Q. Wang, X. J. Chen, Nanoparticles Mediating the Sustained Puerarin Release Facilitate Improved Brain Delivery to Treat Parkinson's Disease. *ACS Appl. Mater. Inter.* **11**, 45276-45289 (2019).

2. H. Li, X. S. Wen, W. Di, A Simple LC-MS/MS Method for Determination of Magnolol in Rat Blood and its Application in a Pharmacokinetic Study. *Arzneimittelforschung-Drug Research* **62**, 83-87 (2012).

3. S. K. Meles, L. K. Teune, B. M. de Jong, R. A. Dierckx, K. L. Leenders, Metabolic Imaging in Parkinson Disease. *J. Nucl. Med.* **58**, 23-28 (2017).

4. S. Xiong, Z. Li, Y. Liu, Q. Wang, J. Luo, X. Chen, Z. Xie, Y. Zhang, H. Zhang, T. Chen, Brain-targeted delivery shuttled by black phosphorus nanostructure to treat Parkinson's disease. *Biomaterials* **260**, 120339, (2020).
